# Flow Cytometric Immunophenotyping Is Sensitive for the Early Diagnosis of *De Novo* Aggressive Natural Killer Cell Leukemia (ANKL): A Multicenter Retrospective Analysis

**DOI:** 10.1371/journal.pone.0158827

**Published:** 2016-08-02

**Authors:** Yi Li, Jia Wei, Xia Mao, Qingping Gao, Longlong Liu, Ping Cheng, Limei Liu, Xinhua Zhang, Ke Zhang, Jin Wang, Li Zhu, Jianfeng Zhou, Yicheng Zhang, Li Meng, Hanying Sun, Dengju Li, Mei Huang, Wei Huang, Jinniu Deng, Donghua Zhang

**Affiliations:** 1 Department of Hematology, Tongji Hospital, Tongji Medical College, Huazhong University of Science and Technology, Wuhan, China; 2 Department of Hematology, Renmin Hospital of Wuhan University, Wuhan, China; 3 Department of Hematology, Wuhan General Hospital of Guangzhou Military, Wuhan, China; 4 Department of Hematology, Wuhan Integrated Traditional China Medicine and Western Medicine Hospital, Wuhan, China; Karolinska Institutet, SWEDEN

## Abstract

Aggressive natural killer cell leukemia (ANKL) is a fatal hematological neoplasm characterized by a fulminating clinical course and extremely high mortality. Current diagnosis of this disease is not effective during the early stages and it is easily misdiagnosed as other NK cell disorders. We retrospectively analyzed the clinical characteristics and flow cytometric immunophenotype of 47 patients with ANKL. Patients with extranodal NK/T cell lymphoma, nasal type (ENKTL) and chronic lymphoproliferative disorder of NK cell (CLPD-NK), who were diagnosed during the same time period were used for comparisons. Abnormal NK cells in ANKL were found to have a distinctiveCD56bright/CD16dim immunophenotype and markedly increased Ki-67 expression, whereas CD57 negativity and reduced expression of killer immunoglobulin-like receptor (KIR), CD161, CD7, CD8 and perforin were exhibited compared with other NK cell proliferative disorders (p<0.05). The positive rates of flow cytometry detection (97.4%) was higher than those of cytomorphological (89.5%), immunohistochemical (90%), cytogenetic (56.5%) and F-18 fluorodeoxyglucose positron emission tomography/computer tomography (18-FDG-PET/CT) examinations (50%) (p<0.05). ANKL is a highly aggressive leukemia with high mortality. Flow cytometry detection is sensitive for the early and differential diagnosis of ANKL with high specificity.

## Introduction

Aggressive natural killer cell leukemia (ANKL) is a rare type of hematological neoplasm characterized by monoclonal proliferation of NK cells. Patients with that presents with high fever, hepatosplenomegaly, jaundice and pancytopenia and are characterized by rapid deterioration and a short median survival time of less than two months. The disease is more common in Asia and Latin America than in North America and Europe and affects middle-aged men more frequently than women of the same age[1]. In contrast with the ordinary leukemia, neoplastic ANKL cells are scattered in bone marrow and are morphologically atypical. Previously, diagnosing ANKL mainly depended on a comprehensive integration of clinical manifestations; laboratory test results; and cytomorphological, immunohistochemical, cytogenetic and radiographic analyses, which are time consuming and may not be diagnostically useful prior to the occurrence of a ‘cytokine storm’. Therefore, fast and effective diagnostic approaches are needed for this disease.

NK cells are innate immune cells that lack a specific marker indicative of monoclonal proliferation from reactive status, such as the T cell receptor (TCR) molecules on T cells. Therefore early diagnosis is hampered by difficulty in establishing the clonality of NK cells especially when few neoplastic NK cells are present in bone marrow. Current diagnostic methods, such as laboratory, cytomorphological, immunohistochemical, cytogenetic and radiographic examinations do not allow for adequate assessment of NK cell clonality and these methods tend to delay diagnosis because they require a relatively large number of tested cells or a long processing time. Previous studies have indicated that malignant NK cells in ANKL have a special immunophenotype, as identified by flow cytometry[2–18], but distinguishing ANKL-specific cells from reactive NK cells has not yet been accomplished due to the limited variety of antibodies available for testing (Table 1). A recent European study reported two cases of ANKL with a particular immunophenotype that characterized by the differential expression ofCD56, CD16, CD57, killer immunoglobulin-like receptor (KIR) and killer lectin-like receptor (KLR) compared to normal NK cells[19].The results of the referenced study suggest that flow cytometry can be used to detect ANKL-specific cells with high sensitivity. However, only a limited number of cases were evaluated, which is an obvious disadvantage.

**Table 1:** Previous reports of ANKL.

Country	Year	PatientsNumber	Age median(range)	Sex (male:female)	Characteristics of flow cytometry (positive rate, %)								Mortality (%)	OS, days (median, range)
					CD56	CD16	CD57	CD158	CD7	CD8	CD161	Ki-67		
Japan	1990	4	20(13–30)	1:3	100	50	0	ND	ND	0	ND	ND	100	450(6–960)
China	1997	5	41(37–54)	2:3	100	50	0	ND	ND	ND	ND	ND	100	30(3–42)
Korea	2002	13	42(19–64)	7:6	100	0	ND	ND	0	0	ND	ND	92.3	47(4–163)
Japan	2004	22	42(12–80)	7:15	100	75	13	ND	74	29	ND	ND	95.4	58(1–1170)
Japan	2004	5	59(32–62)	3:2	80	66	25	ND	80	ND	0	ND	80	61(1–742)
Japan	2005	22	41(12–80)	8:14	100	50	0	ND	ND	ND	ND	ND	100	60(ND)
Japan	2005	9	32(6–76)	6:3	100	100	0	ND	88	ND	ND	ND	77.8	90(4–270)
Hong Kong	2005	2	30(21–38)	0:2	100	ND	ND	ND	ND	ND	ND	ND	100	<30(ND)
China	2007	9	45(22–70)	7:2	100	11	ND	ND	44	11	11	ND	100	50(20–220)
Korea	2008	4	55(38–78)	2:2	94	81	100	ND	69	18	ND	ND	100	23(2–120)
Korea	2009	20	44(2–70)	14:6	100	100	ND	ND	50	21	ND	ND	95	48(3–2880)
Japan	2012	3	31(21–76)	1:2	100	ND	ND	ND	ND	ND	ND	ND	100	13(4–140)
China	2013	43	36(15–67)	31:12	100	27	0	ND	47	0	ND	ND	100	22(ND)
China	2013	20	40(17–67)	8:12	100	100	ND	ND	35	ND	ND	ND	100	56(7–343)
China	2014	6	46(37–50)	5:1	100	ND	ND	ND	100	66	ND	ND	83.3	58(-540)
Portugal	2015	2	51(36–65)	1:1	100	50	0	0	50	50	50	ND	100	87(8–365)
China	2015	47	30(14–65)	28:19	97	12	3	4	60	16	48	82	95.7	27(2–1283)

Abbreviations: ND, not determined; OS, overall survival. Mortality was collected on the last follow up before the paper published.

In the current study, we performed a fairly comprehensive analysis of clinical information related to flow cytometric immunophenotype and laboratory, cytomorphological, immunohistochemical, cytogenetic and radiographic examinations of neoplastic NK cells in 47 patients with ANKL. In addition, we compared ANKL with different types of NK cell disorders including extranodal natural killer/T cell lymphoma, nasal type (ENKTL) and chronic lymphoproliferative disorder of NK cells (CLPD-NK) for differential diagnosis according to the World Health Organization (WHO) 2008 classification of NK cell neoplasms[20–22].

## Methods

### Patients and clinical characteristics

A total of 47 consecutive patients were diagnosed with ANKL between January 2008 and January 2015 at four clinical centers in Wuhan, China. The diagnostic criteria were based on published criteria[6, 20]. An initial diagnosis was made at each center and was later reviewed if the original pathological materials were available. Patients were excluded if they meet one of the following conditions: 1. primary or concurrent nasal lesions; 2. CD3 positive status, as detected by flow cytometry or rearrangement of TCR genes; 3. positivity for the AML1/ETO, BCR/ABL, PML/RARα or CBFβ/MYH11 genes. In total, 27 patients with ENKTL and 9 patients with CLPD-NK who were diagnosed according to published criteria[21, 22] within the same period were retrospectively analyzed. 15 healthy volunteer donors were included as a control group. Informed consent was obtained from each participant in agreement with the Declaration of Helsinki and the study was approved by the central institutional review boards of the Ethics Committee of Tongji Hospital prior to study initation. All patients were followed-up at the clinic or via a telephone call through February 2015. The median follow-up time was 27 days (2–1283 days).

Patient clinical information of was reviewed in detail. Additionally, dynamic changes in laboratory test results were monitored during hospitalization. The level of Epstein-Barr virus(EBV)DNA was determined by real-time quantitative polymerase chain reaction (PCR) using a LightCycler EBV quantification kit as previously described [23].The levels of cytomegalovirus(CMV) DNA;(herpes simplex virus)HSVⅠandⅡDNA; and (human herpes virus)HHV6, 7 and 8DNA were tested using real-time quantitative PCR. CMV IgG and IgM levels were measured via enzyme-linked immunosorbent assay(ELISA) as previously described[24].

### Flow cytometry

Bone marrow aspirations and peripheral blood samples were obtained from all the 47 patients. Lymph node specimens were obtained from 20 patients, and a spleen specimen was obtained from one patient. All obtained patient samples were subjected to flow cytometric examination. Lymphocytes or abnormal cell populations were gated in aCD45/linear side scatter (SSC) histogram or in a linear forward-angle light scatter/SSC histogram. Antibodies against the following proteins were used to identify abnormal NK cell phenotypes:, the NK cell surface markers CD56(Clone: B159), CD16 (Clone: 3G8)and CD57(Clone: NK-1); the NK cell surface receptors CD158a/h(Clone: HP-3E4), CD158b(Clone: CH-L), CD158e(Clone: DX9), CD94 (Clone: HP-3D9)and CD161(Clone: DX12); and the T cell-associated antigens CD2(Clone: S5.2), CD3(Clone: UCHT1), CD4(Clone: RPA-T4), CD5(Clone: UCHT2), CD7(Clone: M-T701), CD8(Clone: RPA-T8), TCRαβ(Clone: T10B9.1A-31)and TCRγδ(Clone: B1), perforin(Clone: δG9), granzyme B(Clone: GB11) and Ki-67(Clone: B56). All antibodies except for CD158ewere purchased from BD Diagnostics (Shanghai, China). And anti-CD158e antibody was from Miltenyi Biotec(Shanghai, China). Immunophenotypes were detected using multiparameter flow cytometry (FACSCanto, Becton Dickinson, USA). Data were analyzed using BD FACSDiva software. The strength of antigen expression was measured depending on the mean fluorescence intensity (MFI). To ensure instrument stability, flow cytometry parameters were monitored and set using BD CaliBREADS 3 and CaliBREADS APC beads (BD Diagnostics, USA).

### Bone marrow cytomorphology and immunohistochemistry

The bone marrow aspiration and peripheral blood smears specimens were subjected to Wright-Giemsa staining, and the bone marrow biopsies were embedded in paraffin and stained with hematoxylin-eosin. Antibodies for immunohistochemistry were obtained from Gene Tech (Shanghai, China), for the following proteins: CD56, CD3, CD19, CD20, CD79a, TdT, CD 43 and MPO. An antibody against NKG2A was obtained from Proteintech(Wuhan, China), and an antibody against NKG2C was obtained from Abcam (Cambridge Mass, USA).Double immunofluorescent staining of CD56 (Proteintech, Wuhan, China) and CD3 (Proteintech, Wuhan, China)was also performed.

### Karyotype analysis

Karyotype analysis was performed using a modified chromosome banding technique (G-banding). At least 20 metaphase chromosomes were counted in each sample by microphotography according to the International System for Cytogenetic Nomenclature.

### Statistical analysis

Differences among the immunophenotypes corresponding to the three types of NK cell diseases were analyzed by pairwise comparison (LSD-t test). Overall survival (OS) was estimated using the Kaplan-Meier method and compared by the log-rank test. Factors affecting prognosis were analyzed using the Cox proportional hazard model. Analyses were performed with SPSS13.0. Statistical significance was defined as p<0.05.

## Results

### Clinical, morphological, immunophenotypic, cytogenetic and PET/CT characteristics of ANKL in the cohort

#### Clinical characteristics

The clinical characteristics corresponding to the three categories of NK cell disorders are listed in Table 2. Fever was the most prominent reason cause (68.4%) for admission. In total, 13 patients (27.7%) were originally admitted to another department (non-hematological), delaying their diagnoses by 2–12 days (median, 5 days) before expert treatment. Splenomegaly (89.2%) was the most common symptom. In total, 95.5%of the patients had peripheral blood samples positive for EBV-DNA (>5×10^3 copies/ml).All tested patients were negative for CMV-DNA, CMV-IgM and HSVⅠ andⅡDNA; and HHV6, 7 and 8DNA. A total of 85% of the patients were positive for CMV-IgG.

**Table 2:** The clinical characteristics of ANKL, ENKTL and CLPD-NK, and of EBV+ and EBV- ANKL.

Characteristics	ANKL n (%)	ENKTL n (%)	CLPD-NK n (%)	EBV+ ANKL n (%)	EBV- ANKL n (%)
♢Age(years)	30(14–65)	35(16–72)	37(17–62)	30(14–65)	25(19–30)
Sex(male:female) *^■^	28:19	23:4	5:4	26:19	2:0
♢The highest fever(°C)**^▲▲^	40(36.8–42)	39(36.6–40.2)	37.2(36.8–37.2)	40(36.8–42)	41(41–41)
Hepatomegaly	25(53.2)	4(14.8)	5(55.6)	23(51.1)	2(100)
Splenomegaly	41(87.2)	18(66.7)	2(22.2)	40(88.9)	1 (50)
Mainly involved organ					
Bone marrow	47(100)	12(44.4)	9(100)	45(100)	2(100)
Peripheral blood	27(57.4)	0(0)	9(100)	26(57.8)	1(50)
Lymph gland	19(40.4)	10(37.0)	1(11.1)	19(42.2)	0(0)
Other involvement					
Skin	3(8.1)	4(14.8)	0(0)	3(8.5)	0(0)
Central nervous system	3(8.1)	0(0)	0(0)	3(8.5)	0(0)
Testis	1(2.7)	5(18.5)	0(0)	1(2.8)	0(0)
Lung	1(2.7)	1(3.7)	0(0)	1(2.8)	0(0)
Pancreas	1(2.7)	1(3.7)	0(0)	1(2.8)	0(0)
Nose	0(0)	18(66.7)	0(0)	0(0)	0(0)
Pharynx	0(0)	3(11.1)	0(0)	0(0)	0(0)
IPI score**					
High	37(78.7)	6(22.2)	-	36(80)	1(50)
High intermediate	10(21.3)	7(25.9)	-	9(20)	1(50)
Low or low intermediate	0(0)	14(51.9)	-	0(0)	0(0)
EBV-positive*	42(95.5)	12(60)	5(71.4)	-	-
HLH**^▲▲^^■^	13(27.7)	0(0)	0(0)	13(28.9)	0(0)

Abbreviations: ANKL, aggressive natural killer cell leukemia; ENKTL, extranodal NK/T cell lymphoma, nasal type; CLPD-NK, chronic lymphoproliferative disorder of NK cell; IPI, International Prognostic Index; EBV, Epstein-Barr virus; HLH, hemophagocyticlymphohistiocytosis.

♢The numbers present median (range).

* p<0.05, compared with ENKTL

** p<0.01, compared with ENKTL

^▲▲^p<0.01, compared with CLPD-NK.

^■^p<0.05, compared between EBV+ and EBV- ANKL.

The laboratory results for our cohort are shown in Table 3. At diagnosis, 89.5% of the patients had thrombocytopenia and anemia, and 18.4% had neutropenia (<0.5×10^9/L). In total, 63.2% of the patients had a prolonged prothrombin time (>16 seconds), 57.9% had a prolonged active partial thromboplastin time (>53 seconds), and 63.2% had a decreased fibrinogen level (<2 g/L). Strikingly low level of albumin (<35g/L) (86.8%), increased levels of aspartate aminotransferase (AST) (84.2%) and alanine aminotransferase (ALT) (63.2%) and jaundice (total bilirubin (T-BIL)>17.1μmol/L, direct bilirubin (D-BIL)>6.8 μmol/L) (76.3%) were common. Increased serum creatinine (sCr) (28.9%) was not common. Among the other tested serum markers, lactate dehydrogenase (LDH) was elevated in all patients. Increased levels of β2-MG (87.5%) and ferritin (90.9%) were also common. The patients with ANKL who were EBV+ exhibited higher serum levels of β2-MGand more occurrences of hemophagocytic lymphohistiocytosis (HLH) than the patients with ANKL who were EBV-. Furthermore, CD7expressionwas lower in the EBV+ patients with ANKL compared to the EBV- patients with ANKL (Table 2 and Table 3, S2B Fig). The EBV+ patients with ANKL were positive for NKG2A while the EBV- patients with ANKL were negative.

**Table 3:** The laboratory characteristics of ANKL, ENKTL and CLPD-NK, and of EBV+ and EBV- ANKL.

Characteristics	ANKL Median (range)	ENKTL Median (range)	CLPD-NK Median (range)	EBV+ ANKL Median (range)	EBV- ANKL Median (range)	Normal range
WBC (×10^9/L)	2.8(0.28–61.4)	4.8(0.55–13.8)	3.62(1.01–16.26)	2.8(0.28–61.4)	1.6(1.6–1.61)	4.0–10.0
Hb (g/L) **	83(51–134)	117.5(75–158)	105(41–126)	83(51–134)	66(52–80)	110–160
PLT (×10^9/L) **^▲▲^	28(7.9–337)	177(12–495)	96(30–252)	28(7.9–337)	23.5(19–28)	100–300
PT (s)	15.4(10.8–180)	14.0(11.6–22.2)	13.3(12–15.8)	15.4(10.8–180)	14.9(13.6–16.3)	11.5–14.5
APTT (s)	44.4(22.7–141)	42.1(24.9–75.7)	41.9(31.3–46.7)	44.4(22.7–141)	42.5(39.2–45.7)	29–42
Fibrinogen (g/L) **^▲^	1.59(0.46–5.01)	2.75(1.03–4.87)	2.88(1.24–3.96)	1.59(0.46–5.01)	2.85(2.1–3.67)	2–4
D-dimer (mg/L)^▲^	3.0(0.2–75.56)	0.72(0.2–13.73)	0.40(0.2–0.32)	3.0(0.2–75.56)	0.8(0.2–1.4)	<0.5
Albumin (g/L) **^▲▲^	25.9(15–41.4)	35.1(18.2–47.1)	34.7(23.4–45.9)	25.9(15–41.4)	24.9(24.3–25.5)	35–52
AST (U/L) *	133(4–2513)	37(14–960)	22(10–168)	133(4–2513)	196.5(54–339)	4–40
ALT (U/L)	50(10–1555)	35(14–920)	23(11–86)	50(10–1555)	148.5(59–238)	4–40
T-BIL (umol/L) **	31.4(6–441.41)	10.1(4.1–95)	14.7(5.7–60.9)	31.4(6–441.41)	114.3(14.9–213.6)	3.4–17.1
D-BIL (umol/L) **^▲^	19.2(1.4–366.8)	3.4(1–82.5)	4.3(11.7–16.2)	19.2(1.4–366.8)	80.7(7.8–153.5)	<6.8
sCr (umol/L) *	65(23–382)	59(37–118)	53(44–73)	65(23–382)	58(56–59)	59–104
LDH (U/L) *^▲▲^	978(239–20803)	323(153–3714)	265(85–493)	978(239–20803)	1459(598–2321)	95–200
β2-MG (mg/L)^▲▲^^■^	6.89(2.78–18.22)	3.3(1.41–11.74)	2.82(1.85–4.86)	6.89(2.78–18.22)	3.1(2.94–3.26)	0.8–2.2
Ferritin (ug/L)	6658(184–58300)	1396(743–7758)	362(691–2267)	6658(184–58300)	4012(494–7530)	30–400

Abbreviations: ANKL, aggressive natural killer cell leukemia; ENKTL, extranodal NK/T cell lymphoma, nasal type; CLPD-NK, chronic lymphoproliferative disorder of NK cell; WBC, white blood cell count; Hb, hemoglobin level; PLT, platelet count; PT, prothrombin time; APTT, activated partial thromboplastin time; AST, aspartate aminotransferase; ALT, alanine aminotransferase; T-BIL, total bilirubin; D-BIL, direct bilirubin; sCr, serous creatinine; LDH, lactate dehydrogenase.

* p<0.05, compared with ENKTL

** p<0.01, compared with ENKTL

^▲^p<0.05, compared with CLPD-NK

^▲▲^p<0.01, compared with CLPD-NK

^■^p<0.05, compared between EBV+ and EBV- ANKL.

#### Morphological characteristics

**Fig 1:**
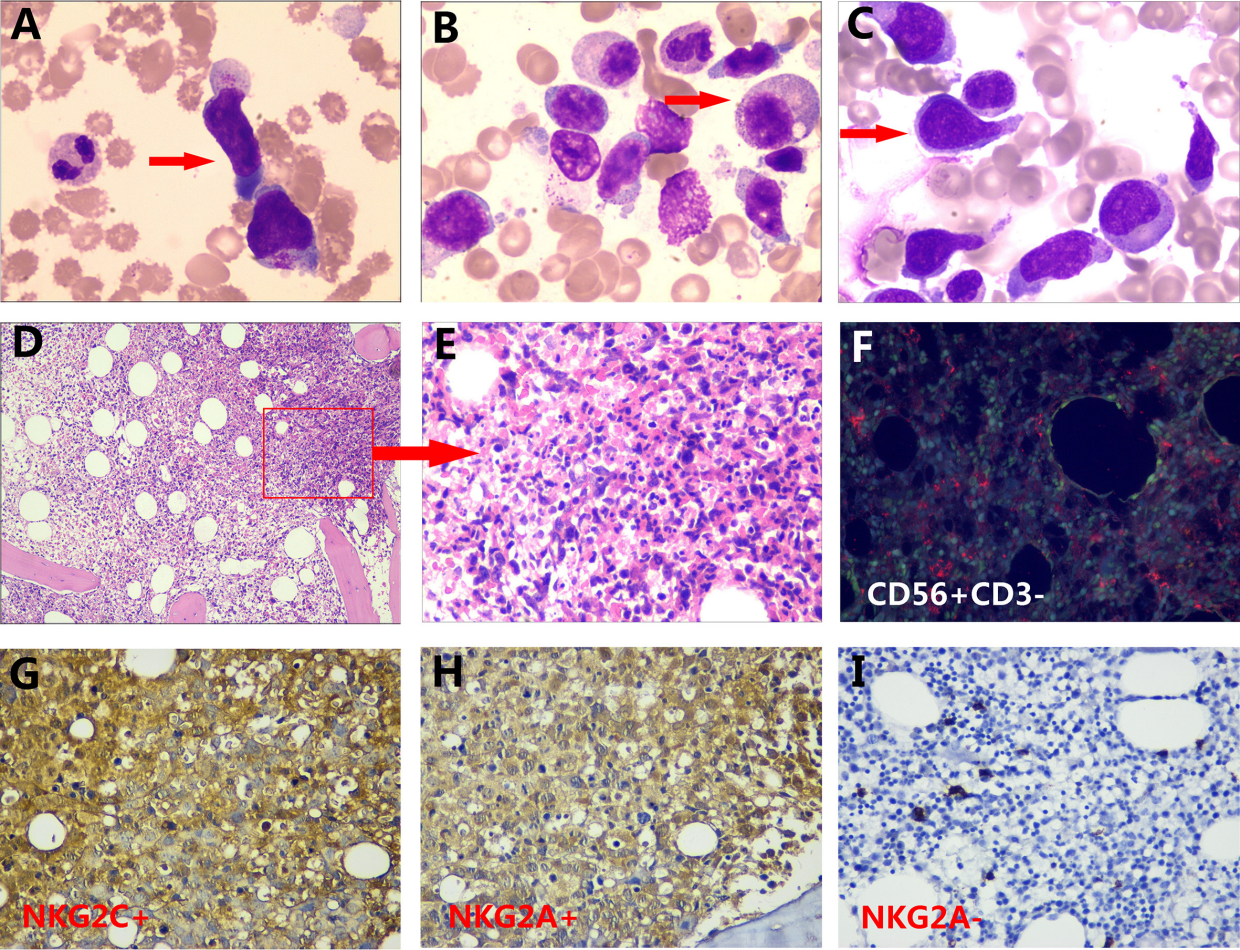
Morphological characteristics of ANKL. (A-C) Large granular lymphocytes, large cells with oval or irregular nuclei and abundant cytoplasm, and coarse azurophilic granules that are clearly visible (red arrow; Wright-Giemsa-stained samples from patients 5, 11 and 36 showed at ×1000). (D, E) Bone marrow biopsy from an ANKL patient showing extensive infiltration of abnormal NK cells (red arrow, from patient 22) (picture E is an enlargement of picture D) (paraffin-embedded, hematoxylin-eosin stained sample from patient 32 showed at ×200,). (F) Double immunofluorescent staining of CD56 (green) and CD3 (red) showingCD56+/CD3- NK cells in one patient’s bone marrow (paraffin-embedded sample from patient 30showed at×400).(G-I) Immunohistochemical results from aberrant NK cells in bone marrow, with NKG2C (brown) positivity(90.5%) (G), NKG2A (brown) positivity (from an EBV+ patient with ANKL) (H) and NKG2Anegativity (from an EBV- patient with ANKL) (I)(paraffin-embedded samples from patient 27, 28 and 29 showed at ×400).

Morphological lymphocyte abnormalities included the presence of large granular lymphocytes, accounting for 20.1%±20.9 of total lymphocytes in the bone marrow (Fig 1). Hemophagocytic phenomena were observed in 28.9% of the patients, and dyserythropoietic phenomena were found in 27.0%. In total, 53.8% of the patients showed infiltration of abnormal cells that were distributed either sporadically (71.4%) or focally (28.6%) in bone marrow biopsies (Fig 1). A total of 90% of the patients submitted to immunohistochemical examination showed a CD56+, CD3-, NKG2A+,NKG2C+ (Fig 1), CD79a-, TdT-, CD 43-,MPO-and CD20-phenotype (S1 Fig).

#### Immunophenotypic characteristics

**Fig 2:**
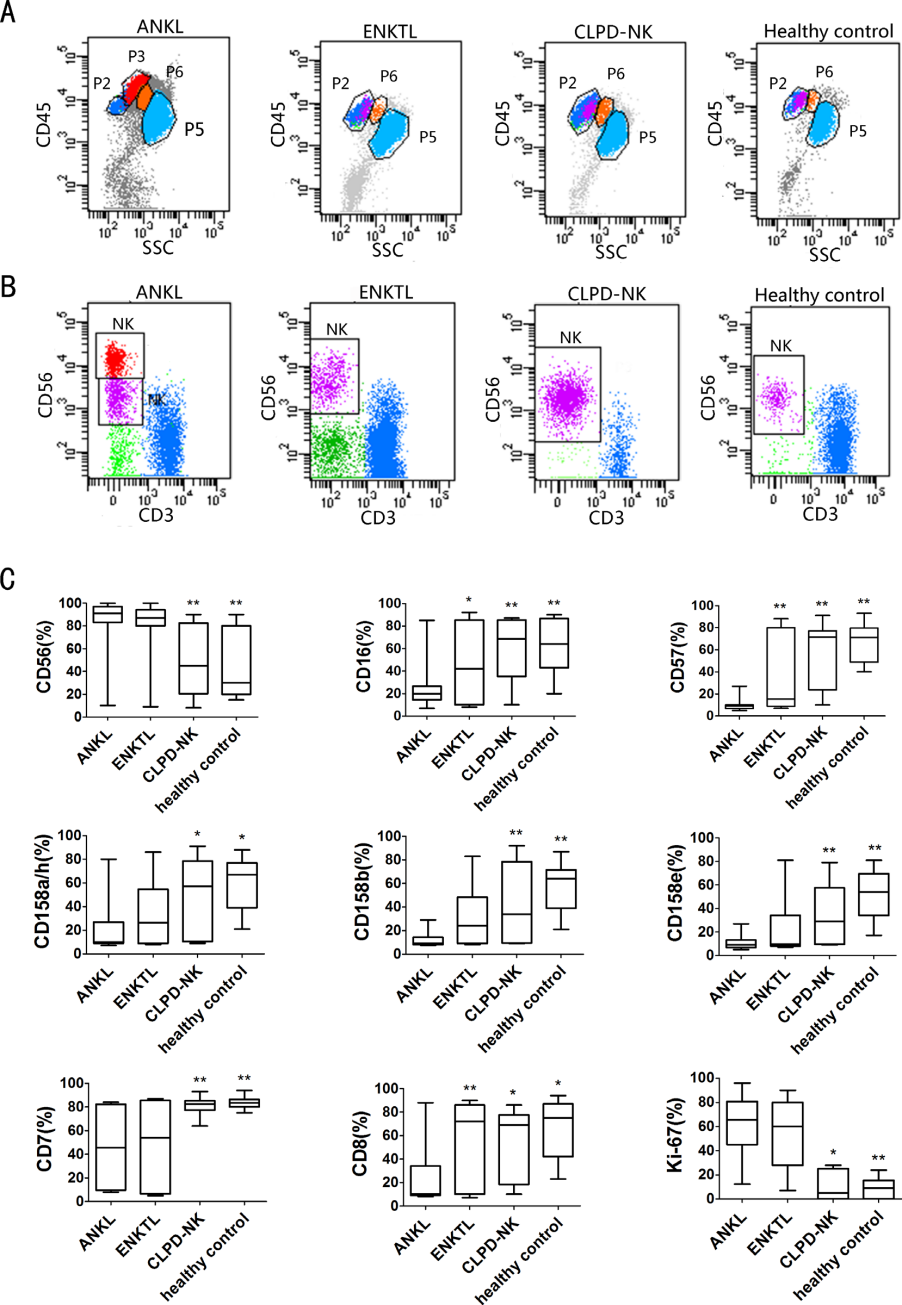
Comparison of immunophenotypic characteristics among samples from different NK diseases and healthy control detected by flow cytometry. (A) Abnormal NK cells from patients with ANKL (red P3 group) were separated from lymphocytes (dark blueP2 group)based on the abnormal expression of the CD45 antigen when aCD45/SSC gating strategy was used. In contrast, in the patients with ENKTL or CLPD-NK and in the healthy controls (purple cell group in P2 group), NK cells usually overlapped with lymphocytes (dark blue P2 group).(B) The NK cells from patients with ANKL could be separated based on aCD56bright (red cell group) and CD56dim (purple cell group) phenotype when a CD56 gating strategy was used. In contrast, in the patients with ENKTL or CLPD-NK and in healthy controls, the NK cells (purple cell group) did not show this phenomenon. (C) The immunophenotypes for the 3 NK cell diseases tested and the healthy controls shown with the extent of expression for each antigen. Abnormal NK cells in patients with ANKL had lower expression levels of CD16, CD57 and CD8 than those from the patients with ENKTL (p<0.05). The expression levels of CD16, CD57, KIR (CD158a/h, CD158b, CD158e), CD7 and CD8 in abnormal NK cells in from the patients with ANKL were significantly decreased compared to those measured in the patients with CLPD-NK; whereas CD56 and Ki-67 were markedly more strongly expressed in the patients with ANKL than in the patients with CLPD-NK.* p<0.05, ** p<0.01.

Infiltration of abnormal NK cells accounted for 19.4%±20.8 of the nucleated cells observed in bone marrow, 20.3%±18.5 of the nucleated cells observed in peripheral blood and 37.4%±24.6 of the nucleated cells observed in lymph nodes. The immunophenotypes of the abnormal NK cells were consistent across sample types (bone marrow, peripheral blood and lymph nodes) (S2A Fig). For approximately half the patients, abnormal cell populations could be separated from lymphocytes when using aCD45/SSC gating strategy, whereas normal NK cells usually overlapped with lymphocytes (Fig 2A). For the remaining patients, abnormal NK cells overlapped with lymphocytes and were identified a CD56/CD3 gating strategy. The expression levels of surface antigens on monoclonal NK cells were defined as stronger, weaker or absent (Fig 2B, 2C and S3 Fig). Several specific characteristics of ANKL were identified in our cohort. First, with regard to NK cell-surface markers, 91.9% of the patients with ANKL had a CD56bright/CD16dim and CD57 negative phenotype. Second, with regard to NK cell receptors, CD158a/h, CD158b and CD158e were entirely lost in 72.7% of the patients and were expressed at fairly low levels in 22.7%. Approximately one third of the patients lacked CD161expression, whereas CD94 expression appeared nearly normal. Third, with regard to T lymphocyte-associated antigens, CD7 and CD8 were frequently absent: 29.7% and 78.4% of the patients, respectively, lacked these markers. CD2 positivity was noted in 97.3% of the patients, whereas CD3, CD4, CD5, TCRαβ and TCRγδ were consistently absent in the total patient population. Fourth, with regard to CD45, CD45RA, CD45RO, and HLA-DR, nearly half of the patients exhibited strong CD45 expression: as a consequence, NK cells could be separated from lymphocytes using aCD45/SSC gating strategy. CD45RA and CD45RO were expressed in half of the patients and HLA-DR was strongly expressed in most patients. Finally, perforin expression was decreased in approximately half of the patients whereas TIA-1 and granzyme B expression remained nearly normal. Ki-67 was heterogeneously expressed at a high level in the majority of the patients, with levels ranging from 0–96.07% (median, 56%).

#### Cytogenetic and PET/CT characteristics of ANKL

**Fig 3:**
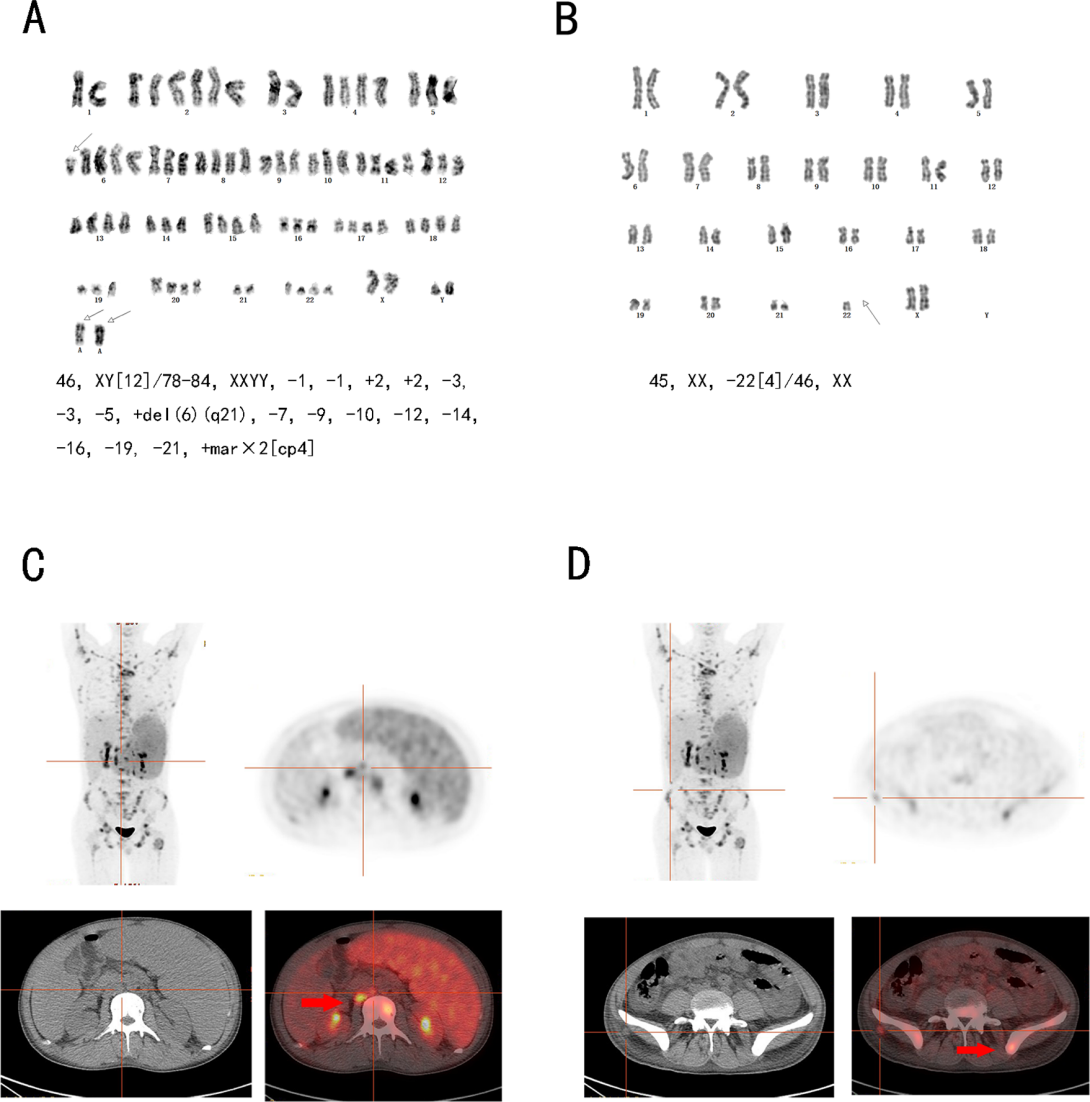
Patients with ANKL had highly complex karyotypes and could be tracked by 18-FDG-PET/CT. (A) Cytogenetic findings for patient 16, who had a hypotetraploid karyotype. (B) Cytogenetic findings of patient 18, who had a karyotype showing the loss of chromosome 22. (C) The 18-FDG-PET/CT result for patient 26 showed splenomegaly and internal lymphadenopathy with uniformly elevated metabolism, and the maximum SUV was 5.6. (D) Patient 26 showed non-uniformly elevated metabolism in the bone marrow, and the maximum SUV was 9.2.

A total of 53.2% of the patients had extremely complex abnormal karyotypes and lacked specificity, 25.5% lacked mitosis and 21.3% were normal. The most common abnormalities involved chromosomes 13 (61.5%) and 11 (38.5%), and others included +8 (23.1%), - 19 (23.1%), - 21 (23.1%), + 1 (15.4%) and + 2 (15.4%) (Fig 3A and 3B). Only half of the patients showed an increase in bone marrow metabolism during 18-FDG-PET/CT examination. A total of 83.3% of the patients displayed increased metabolism in the spleen; however, abnormal NK cells were not found in the one splenectomy specimen obtained. Overall, 50% of the patients showed an increase in metabolism in lymph nodes, but these nodes were positioned too far internally for lymph node biopsy to be performed (Fig 3C).

### Flow cytometry is both sensitive and specific for the early diagnosis of de novo ANKL

Positive rate was used to measure the sensitivities of the different methods employed. The positive rate for bone marrow analysis by flow cytometry immediately after admission was 86.8%. This rate was significantly improved, reaching 94.7%, when bone marrow samples were submitted simultaneously with positive peripheral blood and lymph node samples. The percentage ultimately increased to 97.4% when positive bone marrow samples were collected second and third time several days or weeks after an initial negative examination when the disease developed. The positive rate for morphologically abnormal lymphocytes in the bone marrow was 84.2% for the first analysis, and this rate increased to 89.5% when second and third positive samples were added. We performed immunohistochemical testing along with flow cytometry and found aberrant NK cells, at a positive rate of 90% for the former test. The positive rate for an abnormal cytogenetic karyotype was 53.2%. The positive rate for 18-FDG-PET/CT examination was 50%. We compared the time periods needed to process samples using the above methods and found that, except for morphological examination, the periods of time needed for these methods were prolonged compared to that needed for flow cytometry.

Specificity was compared among the ANKL, ENKTL and CLPD-NK samples by flow cytometry (Fig 2C). The results of other clinical examinations and laboratory tests suggested that the levels of hemoglobin, platelets, fibrinogen, ALB, AST, T-BIL, D-BIL, sCr, and LDH and the rate of EBV-positivity were higher in ANKL than ENKTL (p<0.05). Additionally, the levels of platelets, fibrinogen, D-dimer, LDH and β2-MG were higher for ANKL compared to CLPD-NK (p<0.05) (Table 3). However, these data, only revealed the aggravated clinical profile of ANKL and could not be used to specifically diagnose it. Blastic lymphocytes were also found in the patients with ENKTL or CLPD-NK, but they were difficult to discriminate from ANKL-specific cells because they had similar morphologies. The phenotypes revealed by immunohistochemistry showed no significant differences (p>0.05) among the different types of NK cell diseases. Extremely heterogeneous karyotypes were mainly found in ANKL, and they could not be compared with those of other NK cell disorders. In total, 11.1% of the patients with CLPD-NK displayed elevated metabolism in the spleen and bone via 18-FDG-PET/CT, with a lower standardized uptake value (SUV) than that in the patients with ANKL (p<0.05); but there were too few patients to reach any meaningful conclusion.

### ANKL developed rapidly and had high mortality

**Fig 4:**
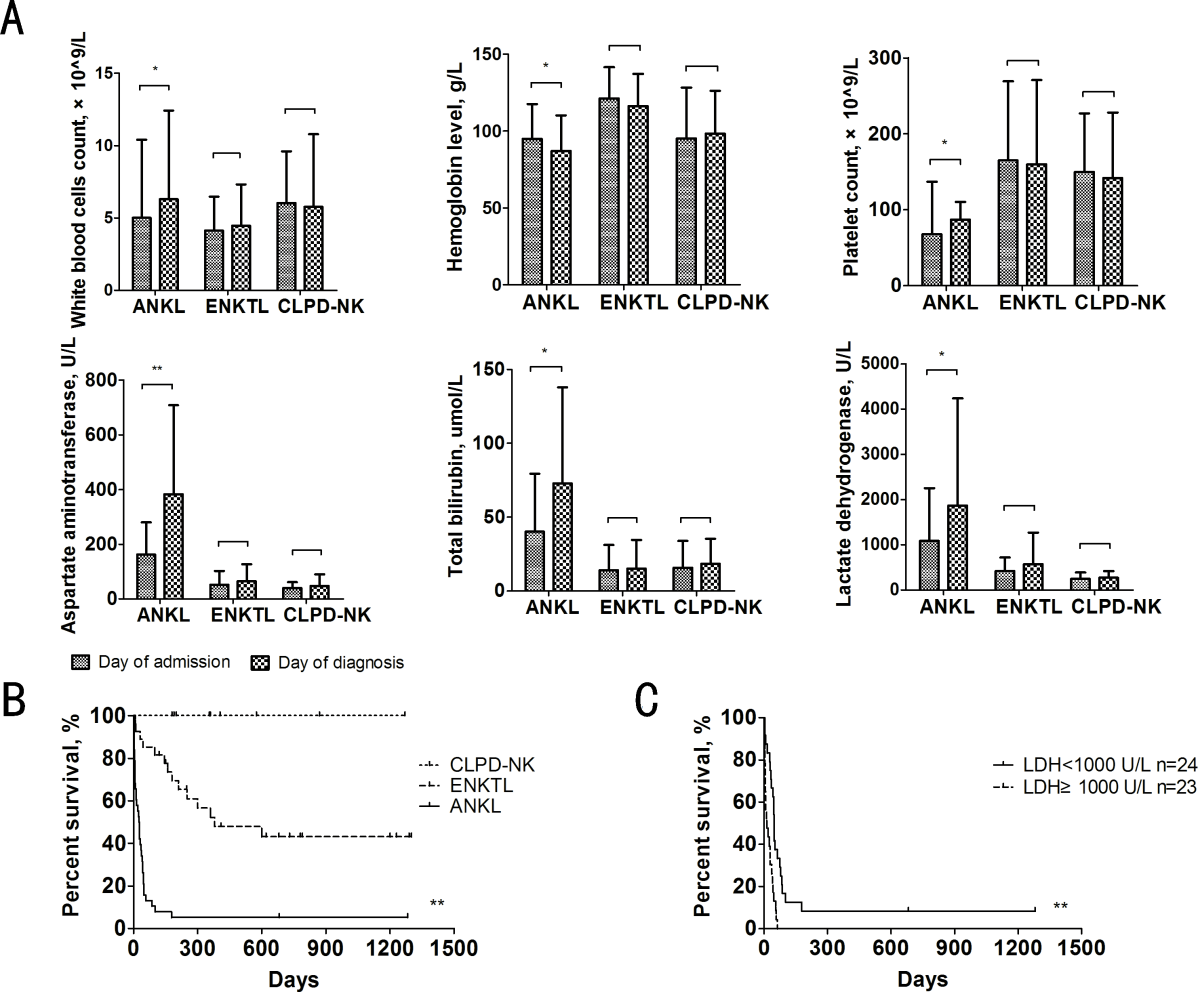
ANKL developed aggressively and had a low OS. (A) Laboratory test results were monitored from the day of admission to the day of diagnosis for all three types of NK cells diseases. The significantly dynamic data reflected the rapid deterioration of the patients with ANKL compared to those with the other two disease types. * p<0.05, ** p<0.01. (B) Prognosis for the three evaluated NK cell diseases, as analyzed by the Kaplan-Meier method. The median OS for ANKL was 27 days (range, 2–1283 days), whereas that for ENKTL was 249 days (range,8–1300 days) and that for CLPD-NK was 360 days (range,180–1270 days). The mortality rate for ANKL, ENKTL, and CLPD-NK were 10.5%, 48.1% and 0% respectively. * p<0.05, ** p<0.01. (C) OS stratification by serum LDH level (＜1000 U/L vs≥1000 U/L) for the patients with ANKL, as analyzed by the Kaplan-Meier method. * p<0.05, ** p<0.01.

The symptoms of and laboratory findings for the majority of the patients indicated irreversible exacerbation (Fig 4A). In this cohort, 52.6% of the patients suffered multiple organ failure and were resistant to chemotherapy. Only two (4.3%) patients achieved complete remission with a regimen that included methotrexate + L-asparaginase + dexamethasone, followed by allo geneic hematopoietic stem cell transplantation (allo-HSCT). These patients were still alive at the last follow-up. The other 45 (95.7%) patients died, with a median survival time of 27 days (2–1283 days) (Fig 4B). The 3-month OS was only 10.6%, and the one-year OS was 4.3%. The most common cause of death was disease progression (63.1%), followed by chemotherapy-associated side effects (31.7%) (such as tumor lysis syndrome or infection). Kaplan-Meier analysis of prognostic factors suggested that a serum LDH level >1000 U/L (p = 0.001), ALB level <25.9 g/L (p = 0.002), AST level >133 U/L (p = 0.001), and D-BIL level >19.2μmol/L (p = 0.029) were poor prognostic factors. The patients with a normal karyotype had a longer OS than those with an abnormal karyotype, but this difference was not significant (p = 0.089). Multivariate Cox regression analysis showed that a serum LDH level >1000 U/L (p = 0.001; relative risk: 4.346; 95%CI: 1.80–10.47) was an independent poor prognostic factor (Fig 4C).

## Discussion

Generally, NK cells could be divided into the following two subtypes according to the strength of CD56 expression: 90% of NK cells were CD56dim/CD16bright, whereas the other 10% were CD56bright/CD16dim. The latter group is thought to represent an earlier stage of NK cell differentiation because the cells in this group had longer telomeres and appeared earlier than those in the former group after allo-HSCT[25, 26]. CD56bright/CD16dim NK cells gradually lose their proliferative capacity and begin to express CD57 and KIR when they differentiate into CD56dim/CD16bright NK cells[27–30]. Consistent with recent studies[16, 19, 31], the abnormal NK cells in 91.9% of the patients with ANKL in our study were CD56bright/CD16dim and CD57 negative, suggesting differentiation failure and an apoptosis disorder. Previous studies have demonstrated that aberrant Notch and STAT signaling inhibit the differentiation and apoptosis of NK cells in association with tumor formation[32–35]. This phenomenon could underline the abnormal immunophenotype noted in ANKL diagnosis in the current study.

The main challenges associated with early diagnosis of ANKL are the small number of abnormal NK cells in the bone marrow and the maldistribution of these cells in this context. In half the patients in the current study, the proportion of abnormal NK cells in the bone marrow was less than 5%. Additionally, we demonstrated that flow cytometry had the highest sensitivity for detecting such low percentages of neoplastic NK cells. Following the identification of abnormal NK cells, confirmation that they were exclusive to ANKL became problematic because atypical NK cells also exist in other NK cell proliferative disorders such as ENKTL and CLPD-NK. Previous studies have identified certain surface antigens with distinct expression profiles among different NK cell diseases, but these studies failed to assess KIR and Ki-67 (Table 1). Our results revealed the poor specificities of cytomorphological, immunohistochemical, cytogenetic and PET/CT examinations, which are inadequate to distinguish ANKL from the other two diseases studied. In contrast, the immunophenotype of ANKL detected by flow cytometry significantly differed from those of CLPD-NK and ENKTL. This relatively high specificity is necessary to establish a diagnosis of ANKL.

In recent years, the importance of NK cell surface major histocompatibility complex-Ⅰ (MHC-Ⅰ) related receptors (KIR and KLR) in differentiating reactive and malignant NK cell proliferative diseases has been demonstrated. Few clinical studies on anti-KIR antibodies in ANKL have been reported to date [36–38]. Normal and polyclonal NK cells usually express a variety of KIR subtypes, whereas monoclonal NK cells exhibit restricted expression of a single KIR isoform or a complete lack of expression. Our cohort exhibited obvious absences of CD158a/h, CD158b and CD158e in NK cells of ANKL. We only tested for the above KIR types due to the limited antibodies currently available for testing. KIRs are the key molecules that recognize MHC class I molecules, which define infected cells and tumor cells [39–42]. One-third of the patients in our study lacked CD7, which is considered to be associated with cellular clonality[13, 16, 19]. The Ki-67 antigen is a nuclear protein whose positivity is correlated with cellular division and proliferation[43]. In the present study, 64% of the patients with ANKL had a Ki-67 expression level higher than 50%, coinciding with the aggressive clinical course of the disease. Perforin expression was lost in 60% of the patients. Damage to the killing function of NK cells, typically mediated by a perforin-dependent pathway, results in ongoing immune activation and the occurrence of a cytokine storm in ANKL. It has been reported that EBV infection induces the expansion of NKG2A+/KIR-/CD56dim NK cells [44],so the NK cells associated with ANKL in the current study may have originated from earlier stage NK cells, as their immunophenotype was NKG2A+/KIR-/CD56bright[45], which favors an EBV origin. CMV can induce the proliferation of NKG2C+/KIR+ cells[46], and it was reported that nearly 20% of patients with CLPD-NK also express NKG2C in association with CMV infection[47]. Because most of the patients in the current study were negative for CMV-DNA and CMV-IgM but positive for CMV-IgG,theNKG2Cpositivity of the abnormal NK cells from the patients with ANKL may have resulted from previous infection of CMV.Moreover, the expression of activating KIR isoforms associated with CMV infection in CLPD-NK[48] is different from the lack of KIR expression in ANKL and may help to identify the two diseases. In comparing EBV+ patients with ANKL to EBV- patients with ANKL, we found that the former had more serious manifestations than the latter. Additionally, there was lower CD7 expression in the EBV+ compared to the EBV- patients. Based on these findings, further research is needed to confirm whether two types of ANKL exist with respect to serum levels of EBV-DNA.

In summary, flow cytometric examination is superior to other clinical tests for ANKL diagnosis in terms of sensitivity and specificity. The strong expression of CD56 and Ki-67 and the weak expression of CD16 CD57, KIR, CD7 and CD8 were useful to differentiate ANKL from CLPD-NK in this study. To enhance sensitvity, specimens could be obtained from multiple regions, such as peripheral blood, enlarged lymph nodes, spleen tissue, rashes and pleural effusions for flow cytometric analysis. As mentioned above, the positive rate eventually rose from 86.8%to 97.4% when second and third positive bone marrow samples were assessed as the disease progressed. Therefore, it is necessary to conduct consecutive monitoring via flow cytometry of samples from feasible regions when positivity is not detected but ANKL is suspected.

Although we established a key role for flow cytometric examination in the early and differential diagnosis of ANKL, other clinical test results should not be disregarded. ANKL patients manifest pancytopenia, which may result from IFN-γ, a negatively regulated cytokine during hematopoiesis that is secreted by CD56bright/CD16dim NK cells. In this study, the patients presented with acute jaundice hepatitis and had elevated in AST and D-BIL, reflecting severe hepatocellular necrosis. This phenomenon may be explained by previous reports showing extensive infiltration of tumor cells in portal and sinusoidal areas in liver biopsies [49] from patients with ANKL as well as increased serum sFasL, which binds to Fas molecules on hepatocyte surfaces, inducing massive hepatocyte apoptosis[50–52]. Abnormal erythropoiesis was observed in one-third of the patients in the present study, concordant with previous reports[18]. This observation may have resulted from compensatory hyperplasia because the patients also had anemia, and no evidence to indicate monoclonal hematopoiesis was found.

Flow cytometric and other clinical examinations should be associated with and complementary to each other, as the latter often provide effective clues for the diagnosis of ANKL. Physicians should not ignore the possibility of ANKL and should order flow cytometry detection when clinical symptoms of a cytokine storm are present, such as a high fever, hepatosplenomegaly and jaundice. Furthermore, the presence of large granular lymphocyte cells found based on morphology suggests that lymphoid antigens (T and NK cells) should be assessed by flow cytometry. Immunohistochemical results can indicate whether aberrant cells are NK-derived, and karyotypic abnormalities lay the foundation for monoclonal proliferation. Additionally, the positive rate can be improved when a puncture or biopsy is performed on lesions with elevated SUV during PET/CT examination [53].

CD7 negativity, an albumin level <26.8 g/L, a Ki-67 positive rate >60%, and EBV positivity have been reported as poor prognostic factors [12, 13, 23, 31]. Somewhat in contrast with these findings, we found that a serum LDH level >1000 U/L, an albumin level <25.9 g/L, AST level >133 U/L, and D-BIL level >19.2 μmol/L are poor prognostic factors; of these, a serum LDH level of >1000 U/L was identified as an independent poor prognostic factor. Importantly, serum LDH should be further explored, not only for diagnosis but also for stratification. No chemotherapeutic regimen is effective for ANKL, although anti-metabolites such as methotrexate and L-asparaginase may improve response rates, and timely chemotherapy followed by allo-HSCT is presently the only curative therapy [54–56].

## Conclusions

The results of this study suggest that ANKL is a highly aggressive leukemia with high mortality. Flow cytometry detection is sensitive for the early and differential diagnosis of ANKL with high specificity. More prospective clinical studies as well as the detection of all KIR subtypes and second-generation gene sequencing of NK cells in ANKL are needed to obtain a better understanding of the pathogenesis of this disease.

## Supporting Information

S1 Fig Immunohistochemical results for aberrant NK cells in bone marrow. Negative staining ofCD79a (A), TdT (B), CD43 (C), MPO (D)and CD20 (E) (paraffin-embedded samples from patients 35–39 shown at ×400).
(TIF)

S2 Fig Comparisons of immunophenotypes among different ANKL samples and between EBV+ and EBV- patients with ANKL. (A) The immunophenotypes of abnormal NK cells were consistent among sample types (i.e., bone marrow, peripheral blood and lymph nodes). No significant differences were found among the three sample types. (B) The immunophenotypes of the EBV+ and EBV- patients with ANKL shown with the levels of expression of CD56, CD16, CD57, CD7, CD8, CD158a/h, CD158b, CD158e, and Ki-67. The expression of CD7 in the EBV- patients with ANKL was higher than in the EBV+ ANKL.* p<0.05.
(TIF)

S3 Fig Immunophenotypic characteristics of ANKL detected by flow cytometry. (A) CD16: Abnormal NK cells in a patient with ANKL (red cell group) showing decreased CD16 expression compared with the NK cells in a healthy control (purple cell group). (B) CD57: Abnormal NK cells in a patient with ANKL (red cell group) showing the absence of CD57expression compared with the CD57 positivity observed in the NK cells from a healthy control (purple cell group).(C) CD7: Abnormal NK cells in a patient with ANKL (red cell group)showing decreased expression of CD7compared with the CD7 positivity observed in the NK cells from a healthy control (purple cell group). (D) Perforin: Abnormal NK cells from a patient with ANKL (red cell group)showing decreased expression of perforin compared with the perforin positivity observed in the NK cells from a healthy control (purple cell group). (E) CD158a/h, CD158b, CD158e: Abnormal NK cells from a patient with ANKL (red cell group)showing the absence of CD158a/h, CD158b, and CD158eexpression compared with the positive expression levels of the molecules observed in the NK cells from a healthy control (purple cell group). (F) Ki-67: Abnormal NK cells from a patient with ANKL (red cell group) showing increased expression of Ki-67 (69.70%)compared with the negative Ki-67 expression (2.4%) observed in the NK cells from a healthy control (purple cell group).
(TIF)
