# Supplementary figures and images for: Flow Cytometric Immunophenotyping Is Sensitive for the Early Diagnosis of De Novo Aggressive Natural Killer Cell Leukemia (ANKL): A Multicenter Retrospective Analysis
Source: PLoS One. 2016 Aug 2;11(8):e0158827. doi: 10.1371/journal.pone.0158827 (PMC4970793; doi:10.1371/journal.pone.0158827)

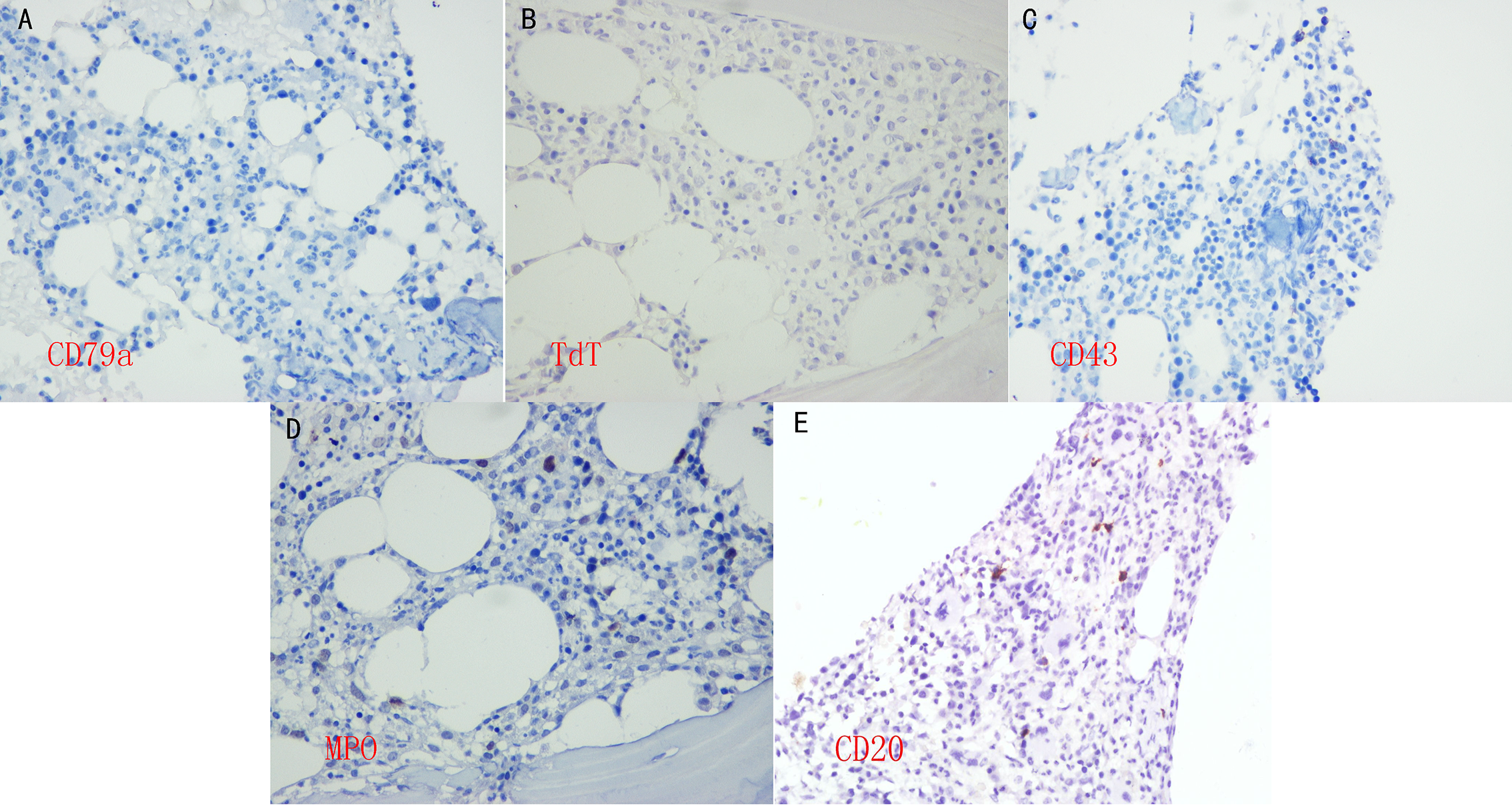

Supplement: S1 Fig — Negative staining ofCD79a (A), TdT (B), CD43 (C), MPO (D)and CD20 (E) (paraffin-embedded samples from patients 35–39 shown at ×400). (TIF) [file pone.0158827.s001.tif]

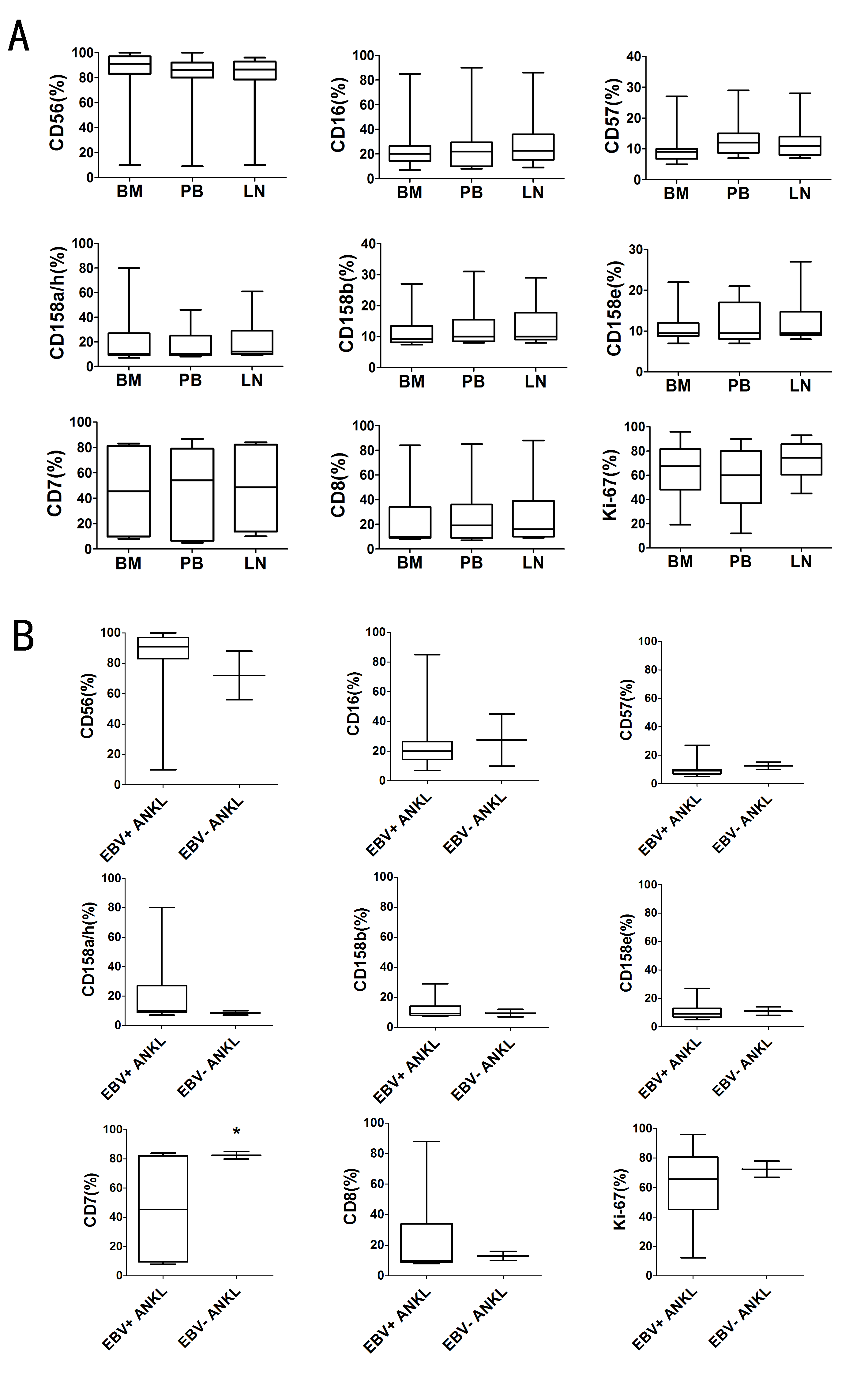

Supplement: S2 Fig — (A) The immunophenotypes of abnormal NK cells were consistent among sample types (i.e., bone marrow, peripheral blood and lymph nodes). No significant differences were found among the three sample types. (B) The immunophenotypes of the EBV+ and EBV- patients with ANKL shown with the levels of expression of CD56, CD16, CD57, CD7, CD8, CD158a/h, CD158b, CD158e, and Ki-67. The expression of CD7 in the EBV- patients with ANKL was higher than in the EBV+ ANKL.* p<0.05. (TIF) [file pone.0158827.s002.tif]

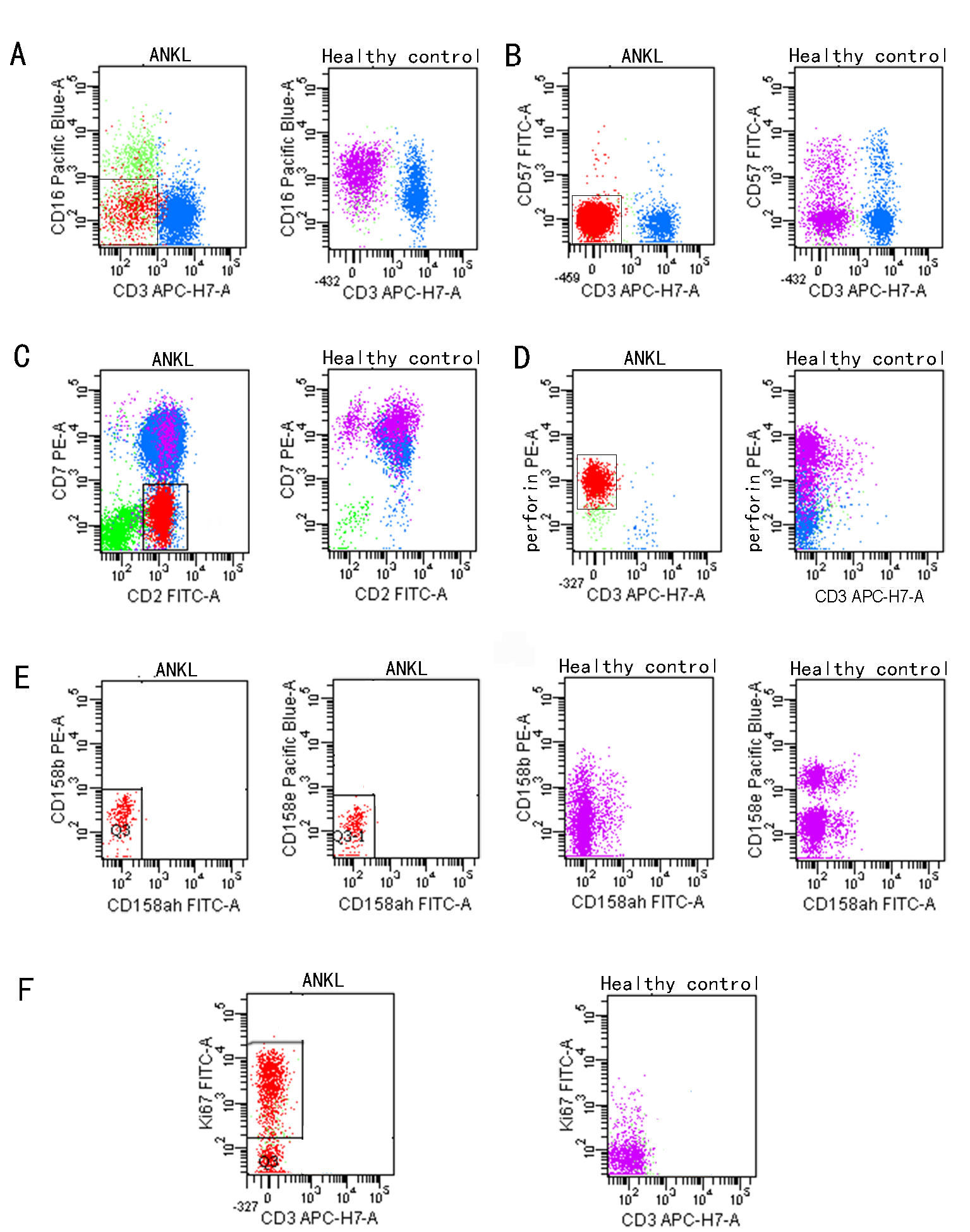

Supplement: S3 Fig — (A) CD16: Abnormal NK cells in a patient with ANKL (red cell group) showing decreased CD16 expression compared with the NK cells in a healthy control (purple cell group). (B) CD57: Abnormal NK cells in a patient with ANKL (red cell group) showing the absence of CD57expression compared with the CD57 positivity observed in the NK cells from a healthy control (purple cell group).(C) CD7: Abnormal NK cells in a patient with ANKL (red cell group)showing decreased expression of CD7compared with the CD7 positivity observed in the NK cells from a healthy control (purple cell group). (D) Perforin: Abnormal NK cells from a patient with ANKL (red cell group)showing decreased expression of perforin compared with the perforin positivity observed in the NK cells from a healthy control (purple cell group). (E) CD158a/h, CD158b, CD158e: Abnormal NK cells from a patient with ANKL (red cell group)showing the absence of CD158a/h, CD158b, and CD158eexpression compared with the positive expression levels of the molecules observed in the NK cells from a healthy control (purple cell group). (F) Ki-67: Abnormal NK cells from a patient with ANKL (red cell group) showing increased expression of Ki-67 (69.70%)compared with the negative Ki-67 expression (2.4%) observed in the NK cells from a healthy control (purple cell group). (TIF) [file pone.0158827.s003.tif]
